# Polymorphic Sites at the 3’ Untranslated Region of the *HLA-G* Gene Are Associated with Differential hla-g Soluble Levels in the Brazilian and French Population

**DOI:** 10.1371/journal.pone.0071742

**Published:** 2013-10-25

**Authors:** Gustavo Martelli-Palomino, Joao A. Pancotto, Yara C. Muniz, Celso T. Mendes-Junior, Erick C. Castelli, Juliana D. Massaro, Irene Krawice-Radanne, Isabelle Poras, Vera Rebmann, Edgardo D. Carosella, Nathalie Rouas-Freiss, Philippe Moreau, Eduardo A. Donadi

**Affiliations:** 1 Program of Basic and Applied Immunology, Faculty of Medicine of Ribeirão Preto, University of São Paulo, São Paulo, Brazil; 2 Commissariat à l’energie atomique et aux energies alternatives, institut des maladies emergentes et des therapies innovantes, service de recherches en hemato-immunologie, Hôpital Saint-Louis, Paris, France; 3 Universite Paris-Diderot, sorbonne paris-cite, umr-e5, institut universitaire d’hematologie, Hopital Saint-Louis, Paris, France; 4 Federal University of Espírito Santo, Sao Mateus, Espirito Santo, Brazil; 5 Program in Cellular and Developmental Biology, Federal University of Santa Catarina, Santa Catarina, Brazil; 6 Departamento de Química, Faculdade de Filosofia, Ciências e Letras de Ribeirão Preto, Universidade de São Paulo, Ribeirão Preto-SP, Brazil; 7 Department of Pathology, School of Medicine of Botucatu, University of the State of São Paulo - UNESP, Botucatu - SP, Brazil; 8 Division of Clinical Immunology, Department of Medicine, School of Medicine of Ribeirao Preto, University of Sâo Paulo, Sâo Paulo, Brazil; 9 Institute for Transfusion Medicine, University Hospital Essen, Essen, Germany; University of Bonn, Institut of experimental hematology and transfusion medicine, Germany

## Abstract

HLA-G molecule has well-recognized tolerogenic properties, and the encoding gene shows lower frequency of polymorphism at the coding region but higher variability at regulatory 5’ and 3’ untranslated (3’UTR) regions. At least three 3’UTR polymorphic sites have been associated with *HLA-G* mRNA regulation, including the 14 base pair (14bp) Insertion/Deletion, +3142C-G and +3187A-G. We studied the association of polymorphic sites at 3’UTR (sequencing analysis, encompassing the 14bp Ins-Del/+3003T-C/+3010C-G/+3027C-A/+3035C-T/+3142C-G/+3187A-G/+3196C-G polymorphic sites) with plasma soluble HLA-G levels (sHLA-G, detected by ELISA) in 187 French and 153 Brazilian healthy individuals. Allele and genotype frequencies were closely similar in both populations; however, Brazilians showed a higher *HLA-G* 3’UTR haplotype diversity. Considering sHLA-G levels in both populations altogether, individuals presenting 14bp Del/Del showed higher levels compared to 14bpIns/Ins genotype (*P* <0.05); those presenting +3010C/G showed higher levels compared to the +3010C-C genotype (*P*< 0.05); those presenting +3027C-C showed higher levels than the +3027A-A genotype (*P*< 0.05); and those bearing +3035C-C showed higher levels compared to the +3035C-T (P < 0.01) and +3035T-T (*P* < 0.05) genotypes. The analyses of 3’UTR haplotypes showed that UTR-1 (DelTGCCCGC) was associated with higher expression of sHLA-G, whereas UTR-5 (InsTCCTGAC) and UTR-7 (InsTCATGAC) with lower expression and other UTRs (UTR-2/3/4/6) exhibited intermediate levels. Since the differential expression of HLA-G may be beneficial or harmful depending on the underlying condition, the identification of individuals genetically programmed to differentially express HLA-G may help on defining novel strategies to control the immune response against the underlying disorder.

## Introduction

HLA-G is a nonclassical class Ib molecule, first identified on fetal extravillous cytotrophoblast cells, placental macrophages, and mesenchymal chronic villi [1], which has been primarily associated with maternal-fetal tolerance [2]. HLA-G is believed to protect the fetus against trophoblast damage caused by maternal NK [3] and CD8+ T cells [4] during pregnancy [2,4], to prevent proliferation of CD4+ T cells [5], and to tolerize dendritic cells [6]. Seven HLA-G isoforms generated by alternative splicing of the primary transcript may be produced. *HLA-G1* to *-G4* mRNAs encode membrane-bound molecules and *HLA-G5* to *-G7* mRNAs encode soluble forms [7].

To date, the expression of HLA-G1 has been exclusively linked to inhibitory function. Diverse studies have shown that HLA-G1 expression on tumor cells inhibits immune effector cell function through interaction with inhibitory leukocyte receptors. At least two major HLA-G leukocyte receptors have been identified, including immunoglobulin-like transcript-2 (ILT2, also designated as CD85j or LILRB1) and ILT4 (CD85d/LILRB2). While ILT2 is primarily expressed by some NK, T and B cells, and by all monocyte/dendritic cells, ILT4 is myeloid-specific and is primarily expressed by monocyte/dendritic cell lineages [8]. The expression of the additional HLA-G receptor KIR2DL4 is mainly restricted to a CD56^bright^ subset of NK cells, which constitute a minority of peripheral NK cells, but a majority of uterine NK cells [9].

In contrast to the classical *HLA* class I loci, limited *HLA-G* coding region variability has been observed in worldwide populations [10], but a relatively higher degree of variation is observed at the 5’ upstream regulatory region (5’ URR) [11] and at the 3’untranslated region (3’UTR) [12]. The *HLA-G* 3’ UTR contains several regulatory elements [13,14], including polyadenylation signals and AU-rich sequences [15], as well as signals that regulate the spatial and temporal expression of *HLA-G* mRNA [16]. Because genetic polymorphisms observed in the *HLA-G* 3’UTR have been associated with the posttranscriptional control of *HLA-G* expression, this gene segment has been studied in autoimmune [17], chronic inflammatory [18] and chronic infectious diseases [19], in allografting [20] and in several types of cancer [21].

One of the 3’UTR polymorphisms is the presence (insertion - Ins) or absence (deletion - Del) of a 14-base pair (14 bp) fragment, in which the Del-Del genotype has been associated with high expression of *HLA-G* mRNA [22–24], whereas the Ins-Ins genotype has been associated with lower mRNA production [23,24]. Although the 14-bp Ins/Del polymorphism has been associated with the magnitude of HLA-G production [22] and modulation of *HLA-G* mRNA stability [25], the implicated mechanisms have not been elucidated. On the other hand, a fraction of *HLA-G* mRNA transcripts presenting the 14-base insertion can be further processed (alternatively spliced) by the removal of 92 bases from the mature *HLA-G* mRNA [22], yielding smaller *HLA-G* transcripts, reported to be more stable than the complete mRNA forms [25].

Single-nucleotide polymorphisms (SNPs) have also been detected at the *HLA-G* 3’UTR, apparently influencing affinity for several microRNAs and also mRNA stability. Particularly, the +3142 G/C and +3187 A/G polymorphic sites have been associated with mRNA degradation and mRNA stability, respectively [26–28]. The presence of a Guanine at the +3142 position was explored by Tan et al. [28] as a susceptibility marker for bronchial asthma, which may influence *HLA-G* expression by increasing the affinity of this region for the miR-148a, miR-148b and miR-152 microRNAs, decreasing mRNA availability by mRNA degradation and translation suppression [29]. The binding ability of these microRNAs may be potentially influenced by other polymorphic sites present at the *HLA-G* 3’UTR, emphasizing the role of the 14-bp fragment, and SNPs at the +3003, +3010, +3027 and +3035 positions, encompassing a region of 32 nucleotides [26]. The +3187 A/G polymorphism is close to (4-bp upstream) an AU-rich motif and has been associated with decreased *in vitro* mRNA stability, so that the presence of the +3187A allele may lead to decreased *HLA-G* expression [15]. Recent studies have reported that the presence of the +3187 A allele is associated with preeclampsia in a Canadian population [15] and with systemic lupus erythematosus in Northeastern Brazilian patients [30].

The three above-mentioned polymorphic sites associated with HLA-G production might also be associated with each other, indicating that their influence may not be mutually exclusive. It is noteworthy that the 14-bp Ins is usually accompanied by the +3142G and +3187A alleles, both previously associated with low mRNA availability, suggesting that lower mRNA production may also be a consequence of the presence of these polymorphic sites in association with the 14-bp fragment [31].

Although several studies have demonstrated the importance of 3’UTR in the HLA-G expression profile, the associations between *HLA-G* polymorphic sites with soluble HLA-G concentration have primarily focused on the 14 bp Ins/Del polymorphic site or on a few polymorphic sites in small cohorts [32]. To provide novel and further data on the impact of the most frequent variation sites described at the *HLA-G* 3’UTR on the plasma soluble HLA-G (sHLA-G) levels we typed the complete *HLA-G* 3’UTR, defining alleles, genotypes, haplotypes and diplotypes in two distinct Brazilian and French populations. Considering that the differential expression of sHLA-G has been primarily associated with the outcome of allotransplanted organs [33], the identification of individuals genetically committed to produce higher or lower HLA-G levels is quite justifiable and clinically relevant.

## Methods

### Subjects

We evaluated 153 (98 male) Brazilian blood samples obtained from healthy blood donors (mean age = 33.65 SD ±12.01), collected at the University Hospital of the School of Medicine of Ribeirão Preto, University of São Paulo, and 187 (105 male) French blood samples from healthy blood donors (mean age 40.97 ± SD 11.66), collected at the French Establishment of blood collection (EFS) of the Saint-Louis Hospital in Paris. The protocol of the study was approved by Comité de Ética do Hospital das Clínicas da Faculdade de Medicina de Ribeirao Preto da Universidade de Sao Paulo(Protocol # 7075/2010), and all participants gave written informed consent before blood withdrawal

### *HLA*-G 3’ UTR typing

DNA was obtained using a salting-out procedure [34]. *HLA-G* 3’UTR variability was assessed by nucleotide sequence variations from +2945 to +3259 nucleotides, using a methodology and a nomenclature described elsewhere [12]. Briefly, amplification was performed in a final volume of 25 μL containing 1X polymerase chain reaction (PCR) buffer (20 mM Tris–HCl, 50 mM KCl, 1.5 mM MgCl_2_) 0.3 mM of each dNTP, 25 pmol of each primer (HG08F and HG08R), 1.0 unit of Taq DNA polymerase (BioTools, Madrid, Spain) and 50 ng of DNA. The initial denaturation step was carried out at 94° C for 3 min, followed by 40 cycles at 94° C for 30 s, 60° C for 60 s, 72° C for 30 s and by a final extension step at 72° C for 7 min. The amplification product was evaluated using a polyacrylamide gel. PCR products containing the amplified fragment of approximately 350 bp were directly sequenced using the reverse primer employed in the amplification and the Big Dye Terminator kit in an ABI3100 Genetic Analyzer (Applied Biosystems®, Foster City, CA). All polymorphic sites observed at 3’UTR were individually annotated and named according to our previous reports [12,35].

### Soluble HLA-G Quantification

Plasma sHLA-G concentration was evaluated in 259 samples (109 French and 150 Brazilian) by a sandwich ELISA using mAb anti-HLA-G (MEM-G/9 - EXBIO, Czech Republic) and anti-β2-microglobulin (DAKO, Brooklyn, NY) as capture and detection antibodies, respectively [36]. Briefly, high binding microtitration plates (FISHER SCIENTIFIC, Waltham, MA) were coated with MEM-G/9 (10 μg/mL) at 4°C, overnight. After saturating the wells with 300 µL of diluent (DAKO) for 2 hours, and after discarding the diluent, 50 µL 2-fold diluted plasma samples were added and incubated for 2 hours. Wells were then incubated with rabbit-anti-human β2-microglobulin detection antibody (DAKO) for an additional hour. To improve the efficiency of the reaction, 100 μL of horseradish peroxidase enhancer (DAKO) was added and incubated for 1 hour. All incubation steps were performed at room temperature. Each step was followed by 4 washes using a specific washing buffer containing H_2_O, PBS 1X and 0.1% Tween (SIGMA, Saint Louis, MO) and a plate washer (THERMO ELECTRON Corporation, Saint Herblain, France). Finally, wells were incubated with a super-sensitive substrate (tetramethylbenzidine (TMB) in mildly acidic buffer (SIGMA) in the dark for 30 min. After the addition of 1 N HCl, optical densities were measured at 450 nm. This ELISA detected both shedding HLA-G1 and soluble HLA-G5 molecules. All samples were assayed in duplicate, and the total sHLA-G levels were determined from a five-point standard curve, using dilutions of calibrated HLA-G5 as a standard reagent. Results were expressed as ng/mL.

### Statistical analysis

The allelic and genotypic frequencies were estimated with the aid of the Genepop software version 4.0.10 [37], using the exact test with Levene’s correction to calculate the number of expected homozygotes or heterozygotes. The exact test of population differentiation based on allelic and genotypic frequencies between the French and Brazilian populations was also carried out using the Genepop program [37]. Adherences of genotypic proportions to expectations under Hardy-Weinberg equilibrium were tested by the exact test of Guo and Thompson [38] using Genepop 4.0.10 [36]. Linkage disequilibrium (LD) between 3’UTR polymorphic sites was evaluated for each group as well as for the entire population using the Arlequin program version v3.5.1.2 [39].

Given the positive LD between SNPs, but unknown gametic phase, the most likely haplotypes for each sample were determined by two independent computational methods, without taking into account any prior information: (i) the expectation-maximization (EM) algorithm [40] implemented with the PL-EM software [41] and (ii) a coalescence-based method implemented with the PHASE v2 software [42]. Concordant haplotypes according to both methods were submitted to the exact test of population differentiation based on haplotype frequencies between French and Brazilian populations using the Arlequin program.

For the analysis of sHLA-G levels and their respective associations with the 3’UTR genotypes, haplotypes and diplotypes we used nonparametric methods for comparing two sample groups (Mann-Whitney) or three sample groups [Kruskal-Wallis (*P*_*KW*_) followed by the Dunn’s posttest]. Correlations between sHLA-G levels and age and gender were determined using the Spearman rank correlation test. These analyses were performed using SPSS Statistics (17.0.2) (SPSS Software) and GraphPad InStat 3.06 (GraphPad Software). For all instances, *P* values < 0.05 were considered to be significant.

## Results

### Polymorphisms at *HLA*-G 3’UTR

To perform these analyses, we evaluated 340 individuals (153 Brazilian and 187 French subjects) who presented data regarding 3’UTR allele, genotype, haplotype, diplotype. The eight previously reported [12], *HLA-G* 3'UTR polymorphic sites [14-bp Ins/Del (rs1704), +3003 C/T (rs1707), +3010C/G (rs1710), +3027A/C (rs17179101), +3035 C/T (rs17179108), +3142 C/G (rs1063320), +3187 A/G (rs9380142) and +3196 C/G (rs1610696)] were observed in Brazilian and French individuals. No other polymorphic site was observed in this region. The allele and genotype frequencies of the eight *HLA-G* 3’UTR polymorphic sites observed for both populations are shown in Table 1. No significant differences were observed when these frequencies were compared between the two populations. 

**Table 1:** Allele and genotype (14 bp Ins/Del, 3003C/T, 3010C/G, 3027A/C, 3035C/T, 3142C/G, 3187A/G, 3196C/G) frequencies observed at *HLA-G* 3’UTR polymorphic sites in Brazilian and French populations.

**Polymorphism**	**Brazilian (*N*= 153)**	**Frequency (%)**	**French (*N*=187)**	**Frequency (%)**	***P* value**
14 pb					
Ins	132	0.4314	154	0.4118	0.325
Del	174	0.5686	220	0.5882	0.265
Del/Del	59	0.3856	62	0.3311	0.123
Ins/Del	56	0.3666	96	0.5133	0.254
Ins/Ins	38	0.2483	29	0.1567	0.635
**+3003**					
C	26	0.0855	45	0.1203	0.125
T	280	0.9155	329	0.8797	0.163
C/C	1	0.0065	2	0.0106	0.174
C/T	24	0.1568	43	0.2299	0.229
T/T	128	0.8366	142	0.7593	0.358
**+3010**					
C	178	0.5817	198	0.5374	0.251
G	128	0.4183	176	0.4626	0.254
C/C	62	0.4052	57	0.3048	0.892
C/G	54	0.3529	90	0.4812	0.456
G/G	37	0.2418	40	0.2139	0.788
**+3027**					
A	20	0.0686	21	0.0588	0.125
C	286	0.9314	353	0.9412	0.258
A/A	3	0.0025	0	0	-
A/C	14	0.0095	22	0.1176	0.213
C/C	136	0.8895	165	0.8823	0.398
**+3035**					
C	261	0.8497	331	0.8850	0.296
T	45	0.1503	43	0.1150	0.502
C/C	115	0.7516	147	0.7862	0.324
C/T	31	0.2026	37	0.1978	0.425
T/T	7	0.0048	3	0.0160	0.115
**+3142**					
C	121	0.3954	172	0.4599	0.124
G	185	0.6046	202	0.5401	0.231
C/C	30	0.1962	38	0.2032	0.370
C/G	61	0.3986	95	0.5088	0.235
G/G	62	0.4052	54	0.2880	0.143
**+3187**					
A	228	0.7451	280	0.7487	0.231
G	78	0.2549	94	0.2513	0.432
A/A	86	0.5622	113	0.6042	0.543
A/G	56	0.3066	63	0.3368	0.213
G/G	11	0.0082	11	0.0580	0.324
**+3196**					
C	213	0.6961	264	0.7059	0.543
G	93	0.3039	110	0.2941	0.366
C/C	80	0.5228	93	0.4973	0.145
C/G	54	0.3568	78	0.4177	0.512
G/G	19	0.1241	16	0.0850	0.684

Haplotype reconstruction of the 3’ UTR polymorphic sites using the EM and PHASE algorithms agreed in 99.42% of the individuals, and the average probability value for haplotype inference using the EM algorithm was 0.9999, while the haplotype inference for the PHASE algorithm was 0.9972. Ten different *HLA-G* 3’ UTR haplotypes were observed for the whole group of individuals, and were designated as previously described by our group (Table 2). Comparison of the haplotypes defined for the two populations showed that Brazilians exhibited higher haplotype diversity (0.8247 ±0.0102) than the French population (0.8100±0.0094). These populations shared eight haplotypes (UTR-1, UTR-2, UTR-3, UTR-4, UTR-5, UTR-6, UTR-7 and UTR-8) and diverged in two haplotypes (UTR-10 and UTR-13), which were exclusively observed in Brazilians. The exact test of population differentiation based on haplotype frequencies (*P*=0.0298 ±0.0075) showed significant differences in haplotype distribution in the Brazilian and French populations. Only the frequency of the UTR-10 haplotype differed significantly between the Brazilian and French populations (*P*=0.0081; Table 2). 

**Table 2:** Haplotype frequencies observed at the *HLA-G* 3’UTR polymorphic sites (14bp Ins/Del, 3003C/T, 3010C/G, 3027A/C, 3035C/T, 3142C/G, 3187A/G, 3196C/G) in Brazilian and French populations.

**Haplotypes**	**Brazilian (*N*=306)**	**Frequency (%)**	**French (*N*=374)**	**Frequency (%)**	***P* value**
**UTR-1 (DelTGCCCGC)**	78	0.2550	94	0.2510	0.929
**UTR-2 (InsTCCCGAG)**	80	0.2610	108	0.2890	0.439
**UTR-3 (DelTCCCGAC)**	46	0.1500	49	0.1310	0.505
**UTR-4 (DelCGCCCAC)**	26	0.0850	45	0.1200	0.165
**UTR-5 (InsTCCTGAC)**	24	0.0780	21	0.0560	0.278
**UTR-6 (DelTGCCCAC)**	17	0.0560	32	0.0860	0.139
**UTR-7 (InsTCATGAC)**	21	0.0690	22	0.0590	0.636
**UTR-8 (InsTGCCGAG)**	7	0.0230	1	0.0050	0.086
**UTR-10 (DelTCCCGAG)**	6	0.0196	0	0.000	0.008
**UTR-13 (DelTCCTGAC)**	1	0.0033	0	0.000	0.450

### Associations between *HLA-G* 3’UTR polymorphic sites and plasma sHLA-G levels

To perform these analyses, we evaluated 259 individuals (150 Brazilian and 109 French subjects) who presented data regarding 3’UTR allele, genotype, haplotype, diplotype and sHLA-G levels. Soluble HLA-G levels did not fit a Gaussian distribution in the Brazilian (*P* <0.0001) or French (*P* <0.0001) populations or in both populations together (*P*<0.0001). No significant correlations were observed between sHLA-G levels and age of the individuals (rS = 0.0169, *P* =0.8638) or between sHLA-G levels and gender of the individuals (median for men = 23.2 ng/mL and median for women = 25.6 ng/mL; *P* =0.8111). 

Overall, the median sHLA-G levels observed for French (25.2 ng/mL) and Brazilian (26.8 ng/mL) individuals were closely similar (*P* =0.9137), and the frequencies of the eight *HLA-G* 3’ UTR polymorphic sites (14bp Ins/Del, +3003 C/T, +3010 C/G, +3027 A/C, +3035 C/T, +3142 C/G, +3187 C/G and +3196 C/G), taken as alleles or genotypes, were also quite similar for the Brazilian and French populations (all *P*-values higher than 0.05). Taking into account these findings and since the major goal was to determine associations between sHLA-G levels and *HLA-G* genotypes, we considered both populations together, and all the observed associations refer to the population as a whole. 

Considering all *HLA-G* 3’UTR genotypes, the 14-bp Ins/Del, +3010C/G, +3027A/C, +3035C/T, +3142C/G and +3187A/G polymorphic sites influenced HLA-G expression (*P*_*KW*_ < 0.05 for all comparisons). The Dunn’s posttest showed significant differences for all these polymorphisms, except for the +3187A/G site. Individuals presenting the 14 bp Del/Del (median = 27.8 ng/mL) genotype showed higher sHLA-G levels compared to those showing the Ins/Ins genotype (median = 22.6 ng/mL) (*P*<0.05). Individuals typed as +3010 C/G (median = 27.7 ng/mL) showed higher sHLA-G levels compared to individuals with the +3010 C/C genotype (median = 23.2 ng/mL) (*P*< 0.05). Individuals presenting the+3027C/C genotype (median = 26.8ng/mL) showed higher soluble HLA-G levels than individuals exhibiting the +3027A/A genotype (median = 11.4 ng/mL, *P* < 0.05). Individuals presenting the +3035 C/C genotype (median = 27.7 ng/mL) showed higher sHLA-G levels compared to individuals with the +3035T/T (median = 14.9 ng/mL) (*P*< 0.05) and +3035 C/T (median = 22.3, *P*< 0.01) genotypes. Individuals presenting the +3142 C/G genotype (median = 27.0 ng/mL) showed higher sHLA-G levels than individuals with the +3142 G/G genotype (median = 23.2 ng/mL) (*P* <0.05).These results are shown in Table 3.

**Table 3:** Comparisons of plasma soluble HLA-G levels (ng/mL) in the whole group of healthy individuals (Brazilian plus French individuals), stratified according to the *HLA-G* 3’ UTR genotypes.

**Polymorphism**	**Soluble HLA-G levels**						Kruskal-Wallis (*P*)^*^
	**Median**		**median**		**median**		
**14-bp**	Del/Del (*n*=94)		Del/Ins (*n*=109)		Ins/Ins (*n*=56)		
	27.8		25.9		22.6		**0.018 ^1^**
**+3003**	C/C (*n*=2)		C/T (*n*=47)		T/T (*n*=210)		
	22.6		25.9		26.5		0.873
**+3010**	C/C (*n*=92)		C/G (*n*=109)		G/G (*n*=58)		
	23.2		27.7		26.4		**0.0162**
**+3027**	A/A (*n*=3)		A/C (*n*=30)		C/C (*n*=226)		
	11.4		22.9		26.8		**0.0063**
**+3035**	C/C (*n*=193)		C/T (*n*=56)		T/T (*n*=10)		
	27.7		22.3		14.9		**<0.0014**
**+3142**	C/C (*n*=49)		C/G (*n*=118)		G/G (*n*=92)		
	27.4		27.0		23.2		**0.0435**
**+3187**	A/A (*n*=148)		A/G (*n*=92)		G/G (*n*=19)		
	25.0		27.3		30.4		**0.032 ^6^**
**+3196**	C/C (*n*=135)		C/G (*n*=96)		G/G (n=28)		
	26.1		25.6		29.9		0.288

Comparisons between the three genotypes.

^1-6^ *P* values as determined by the Dunn’s posttest

**^1^ 14-pb    Del/Del x Ins/Ins *P*< 0.05**

Del/Del x Del/Ins *P*> 0.05

Del/Ins x Ins/Ins *P*> 0.05

**^2^ +3010 C/G**    C/C x GG *P*> 0.05

**C/G x CC *P*< 0.05**

C/G x GG *P*> 0.05

**^3^ +3027A/C    A/A x C/C *P*<0.05**

A/A x A/C *P*> 0.05

A/C x C/C *P*> 0.05

**^4^ +3035 C/T    C/C x T/T *P*< 0.05**

**C/C x C/T *P*< 0.01**

C/T x TT P> 0.05

**^5^ +3142 C/G**    C/C x G/G *P*> 0.05

**C/G xG/G *P*< 0.05**

C/G x G/G *P*> 0.05

**^6^ +3187 A/G**    A/A x GG *P*> 0.05

A/A x A/G *P*> 0.05

A/G x G/G *P*> 0.05

**Figure 1:**
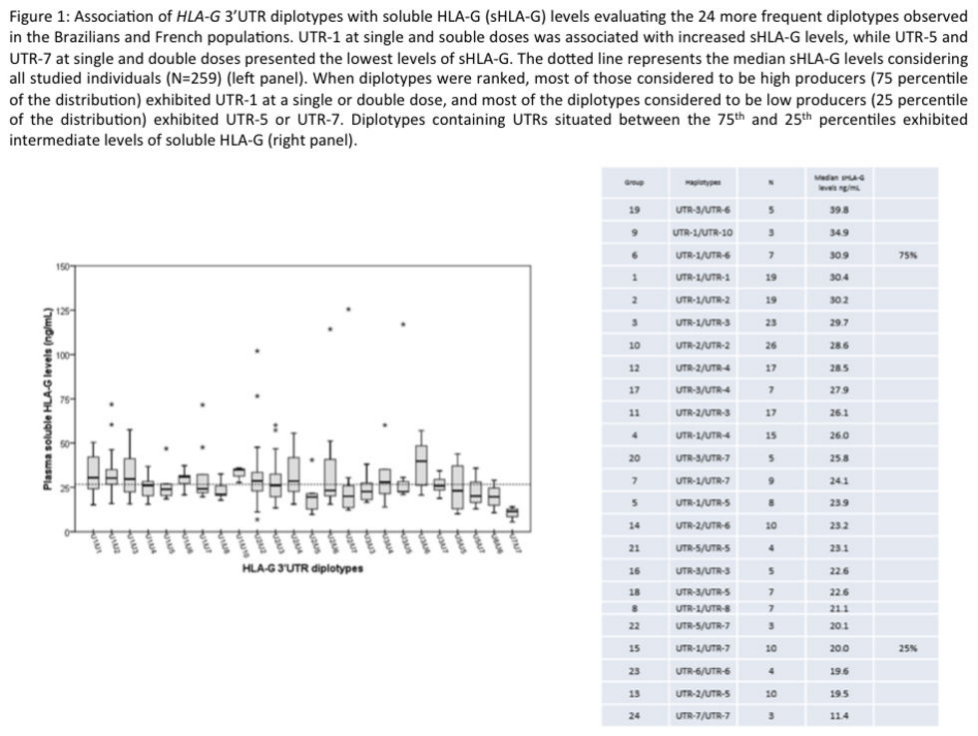
Association of *HLA-G* 3’UTR diplotypes with soluble HLA-G (sHLA-G) levels evaluating the 24 more frequent diplotypes observed in the Brazilians and French populations.

The analysis of the association between sHLA-G levels and 3’ UTR haplotypes was performed using several approaches. Individuals were primarily clustered according to their diplotypes, yielding 24 groups that reached at least 3 observations, as shown in Figure 1. The following analyses were performed:

- comparison of the median values of the 24 different diplotypes, showing significant differences (*P*_*KW*_ = 0.0098);
- Individuals presenting a given UTR were compared according to their second UTR (UTR-1/UTR-1 versus UTR-1/UTR-2 versus UTR-1/UTR-3 versus UTR-1/UTR-4 versus UTR-1/UTR-5 versus UTR-1/UTR-6 versus UTR-1/UTR-7 versus UTR-1/UTR-8 versus UTR-1/UTR-10). This analysis assumed that the given UTR resulted in a basal HLA-G expression level, and the differences between categories (diplotypes) were due to the accompanying UTR. An overall significant probability was observed for UTR-2 (*P*_*KW*_ = 0.0123), revealing that the UTR-2/UTR-1 (median = 30.2 ng/mL) exhibited increased sHLA-G levels compared to the UTR-2/UTR-5 (median = 19.5 ng/mL; *P*< 0.05). No significant differences were observed for UTR-1 and UTR-3 (*P*_*KW*_ = 0.1688 and *P*_*KW*_ = 0.6403, respectively). For the remaining UTRs, this analysis was not performed due to the small number of diplotype groups;
- due to the high frequencies and structural differences between UTR-1 and UTR-2, individuals were compared according to the three different diplotypes involving these UTRs (UTR-1/UTR-1 versus UTR-1/UTR-2 versus UTR-2/UTR-2); showing no significant differences (*P*_*KW*_ = 0.6597);
- comparison of individuals homozygous for different UTRs (UTR-1/UTR-1 versus UTR-2/UTR-2 versus UTR-3/UTR-3 versus UTR-5/UTR-5 versus UTR-6/UTR-6 versus UTR-7/UTR-7) showed significant differences (*P*_*KW*_ = 0.0287); however, the posttest revealed no significant differences;
- the 24 groups were ranked according to the median expression levels, permitting the establishment of three groups of diplotypes defined by the 25^th^ percentile (lower 4^th^ of the distribution, encompassing lower median sHLA-G levels), 75^th^ percentile (upper 4^th^ of the distribution, encompassing higher median sHLA-G levels), and diplotypes situated in between the 25^th^ and 75^th^ percentiles (intermediate 2^nd^ and 3^rd^ fourths of the distribution). The first group (25^th^ lower percentile) was composed mainly by diplotypes containing UTR-5 or UTR-7, while the upper one (75^th^ higher percentile) was mainly composed by diplotypes containing UTR-1. Diplotypes situated in between the 25^th^ and 75^th^ percentiles were composed of several combinations of UTR haplotypes, mainly represented by UTR-2, UTR-3, UTR-4 and UTR-6 (Figure 1). The comparison between each of these percentiles revealed that 75^th^ percentile diplotypes exhibited higher levels compared to 25^th^ percentile diplotypes (*P*< 0.001) and compared to the 25^th^-75^th^ percentile (*P*< 0.05), and the 25^th^-75^th^ percentile presented higher levels than the 25^th^ percentile (*P*< 0.001);
- individuals were grouped and compared according to the presence or absence of a given haplotype in homozygosis or heterozygosis (UTR-1/UTR-1 and UTR-1/UTR-X versus UTR-Z/UTR-W). Diplotypes presenting UTR-1 were associated with increased sHLA-G levels (*P* = 0.0140), and diplotypes presenting UTR-5 or UTR-7 were associated with decreased sHLA-G levels (*P* = 0.0023 and *P* = 0.0213, respectively). For UTRs 2, 3, 4, 6, 8 or 10, no significant differences were found regarding sHLA-G expression.

Therefore, considering all these analyses of sHLA-G levels, we classified UTR-1 as a high producer, UTR-2, UTR-3, UTR-4 and UTR-6 as medium producers, and UTR-5 and UTR-7 as low producers.

## Discussion

Since the *HLA-G* gene has a limited polymorphism at the coding region, relatively few distinct molecules are coded, which present little amino acid variability in protein regions responsible for molecule dimerization and interaction with inhibitory receptors [31]. Consequently, the regulatory regions of the gene have a crucial role in determining the magnitude of gene and protein expression, and polymorphic sites observed along the gene regulatory regions, including the 5’untranslated regulatory region (URR) and 3’UTR, are potential candidates. Little is known about the mechanisms that regulate *HLA-G* expression [31,43] and only a few studies have evaluated the influence of the polymorphic sites seen in the regulatory regions on the magnitude of the soluble levels of HLA-G. In this study, we focused on the association between *HLA-G* 3’ UTR polymorphic sites and plasma levels of sHLA-G. 

Our group previously described the structure of *HLA-G* 5’URR [11] and 3’ UTR in Brazilians [12]. Regarding the 3’UTR, we reported the frequencies of the three polymorphic sites already associated with posttranscriptional control of the gene, including the 14 bp Del/Ins, +3142C/G and +3187A/G. Besides these polymorphisms, we also reported the frequencies of five other SNPs (+3003T/C, +3010C/G, +3027C/A, +3035C/T and + 3196C/G), which have not yet been studied in relation to their influence on the posttranscriptional activity of the gene. This set of polymorphic sites was associated with eight combinations (haplotypes) exhibiting frequencies higher than 1% (UTR-1 to UTR-8) and three other with lower frequencies (UTR-9 to UTR-11) [12,44]. In contrast, there is no systematic survey regarding the structure of the whole *HLA-G* 3’UTR in the French population, except for two previous reports that presented frequencies for the 14bp Ins/Del and +3142, +3187 and +3196SNPs [45,46].

Interestingly, although the French and Brazilian populations have distinct ancestry history, we found no significant differences in the frequencies of the *HLA-G* 3' UTR alleles and genotypes between the two populations. Indeed, the frequency of the 14 bp Ins allele was closely similar for Brazilian (43%) and French (41%) healthy individuals. These frequencies are also similar to those reported for Southeastern (41%) [12], Southern (39.5%) [47], Northeastern (37%) [44], and Native American (38.4%) [48] Brazilians. Quite closer frequencies were observed for Italian (38%) [49], German (38%) [50] and Portuguese (47%) [51] healthy individuals. Although the frequencies of the 14-bp Ins/Del genotypes were quite similar for Brazilian and French individuals, in Brazilians they may vary on a regional basis. In the Northeastern Brazilian population, the frequencies of the Ins/Ins genotype are higher (40.64%) than those of the Southeastern Brazilians of this study (24.83%). In Southern Brazil, the Western European gene pool has a powerful influence, as opposed to the African American gene pool in the Northeastern population [44]. The frequencies of the two other polymorphic sites previously reported to influence *HLA-G* mRNA production, particularly the +3142 G and +3187 A alleles, were quite similar in Southern and Northeastern Brazilians [44], as well as in the French population of the present study; however, allele frequencies may also vary on a regional basis in France [46]. Regarding other polymorphic sites that have not been studied in relation to their influence on HLA-G production, there are no worldwide systematic population studies so far.

Despite the similarity of allele and genotype frequencies, the haplotype diversity varied between the Brazilian and French populations, showing an expected higher diversity in Brazilians, probably due to a more intense rate of miscegenation for Brazilians. Indeed, the *HLA-G* 3’UTR haplotype diversity in Brazilians has shown interesting findings; i.e., a higher haplotype diversity in the Northeastern populations, including rare haplotypes such as UTR-13, UTR-14, UTR-15 and UTR-16 [44] and low diversity for Amerindians from the Amazon Basin (Cagnin et al, unpublished data). The eight more frequent 3’UTR haplotypes were also observed for Northeastern [44] and Southeastern Brazilians as well as for the French individuals of the present study, and for Southern French individuals as reported in the literature [46].

Considering that (i) the frequencies of the eight *HLA-G* 3’ UTR polymorphic sites (14bp Ins/Del, +3003 C/T, +3010 C/G, +3027 A/C, +3035 C/T, +3142 C/G, +3187 C/G and +3196 C/G), taken as alleles or genotypes, were quite similar for the Brazilian and French populations (all *P*-values higher than 0.05), (ii) the most frequent 3’UTR haplotypes (UTR-1 to UTR-8) were quite similarly represented in the Brazilian and French populations, (iii) only few 3’UTR polymorphic sites have been associated with sHLA-G levels in population studies, we pooled Brazilian and French individuals to study the influence of these most frequent *HLA-G* 3’UTR alleles, genotypes, haplotypes and diplotypes on the plasma levels of sHLA-G. 

We observed that individuals exhibiting the 14 bp Del/Del and 14 bp Ins/Del genotypes exhibited higher soluble levels of HLA-G compared to the 14-bp Ins/Ins genotype, reaching significance only for the 14 bp Del/Del. These results corroborate those already described in the literature, evaluating only this polymorphic site [52]. The 14 bp Ins allele (5'-ATTTGTTCATGCCT-3') has been associated with low expression of *HLA-G* and low production of most mRNA isoforms for soluble and membrane-bound molecules [24]. Among the mechanisms proposed to explain these findings, it is of note that the insertion of 14 bases may yield the cutting of 92 bases in a fraction of the primary transcript, eliminating at least two polymorphic sites in the *HLA-G* 3’ UTR and giving rise to shorter mRNAs with increased stability [22,25]. The loss of 92 bases of the primary transcript eliminates a region that may be an important target for microRNAs, which could bind to and inhibit translation or reduce the stability of mRNA. An *in silico* study reported that the deletion of 92 bases, in addition to causing loss of the region of 14 bases, which targets the miR-1229, mir-616, mir-589* microRNAs, also leads to a loss of key regions including the +3003 C/T and +3010 C/G polymorphic sites, which may be targeted by different microRNAs [31]. 

In agreement with the impact of the 14 bp insertion on HLA-G down-regulation, we also observed that individuals exhibiting haplotypes containing the 14 bp insertion, such as UTR-5 (***Ins***TCCTGAC) and UTR-7 (***Ins***TCATGAC) showed lower levels of sHLA-G when compared to subjects exhibiting other UTR haplotypes. On the other hand, most individuals presenting the 14-bp deletion exhibited higher or intermediate levels of sHLA-G, including UTR-1 (***Del***TGCCCGC), UTR-3 (***Del***TCCCGAC), UTR-4 (***Del***CGCCCAC) and UTR-6 (***Del***TGCCCAC), exception made for UTR-2 (***Ins***TCCCGAG) that contains the 14-bp insertion. It should be emphasized that UTR-2 presents other polymorphic sites that are different from UTR-5 and UTR-7, including the +3035C variation site, which was associated with high levels of sHLA-G in the present study. Thus, it is possible that the presence of other variation sites associated with high production may balance the effect of the 14-bp insertion in subjects typed as UTR-2. Although explanations for the mechanisms associated with the role of the Ins/Del polymorphic site are still lacking, this study corroborated, in two different populations, the results that have been previously reported for the German population regarding the role of the 14-bp Ins/Del polymorphism [52].

Despite controversial [28,29], a previous *in vitro* study showed that the presence of a Guanine at position +3142 at the *HLA-G* 3’UTR increased the affinity of the primary transcript to the miR-152, miR-148b, miR-148a microRNAs, inducing degradation of the mRNA or inhibiting translation and, consequently, decreasing *HLA-G* expression [29]. In our genotype/phenotype association study, we showed that individuals exhibiting the +3142 C/C and +3142 C/G genotypes presented higher levels of sHLA-G compared to individuals harboring the +3142 G/G genotype, reaching significance only for the +3142 C/G. Indeed, individuals exhibiting *HLA*-*G* 3’ UTR haplotypes containing the +3142 G allele, including UTR-5 (InsTCCT***G***AC) and UTR-7 (InsTCAT***G***AC) showed lower levels of sHLA-G, and individuals exhibiting high or intermediate levels of soluble HLA-G, including UTR-1 (DelTGCC***C***GC), UTR-4 (DelCGCC***C***AC) and UTR-6 (DelTGCC***C***AC), presented the +3142 C variation site. Exception made for the high *HLA-G* producer haplotypes including UTR-2 (InsTCCC***G***AG) and UTR-3 (DelTCCC***G***AC), which exhibit the +3142 G allele and were classified as intermediate producers. Once again, these findings reinforce the idea that other polymorphic sites present in the 3’UTR may counterbalance the effect of the specific variation site. Alternatively a possible explanation for UTR-2 or UTR3 behavior, considering the presence or absence of the 14 bp deletion or of the +3142 G allele, might be due to the promoter regions specifically associated with these 3’UTR, that may influence mRNA production [11].

The +3187 A/G variation site is located 4 bp upstream of an AU-rich motif that mediates mRNA degradation. An *in vitro* study showed that the presence of the +3187A allele decreased the *HLA-G* mRNA stability, leading to a decreased *HLA-G* expression [15]; however, protein levels or microRNA profiles were not investigated. Our genotype/phenotype association study confirmed that the +3187 A/A genotype was associated with decreased soluble levels of HLA-G compared to individuals exhibiting the +3187 A/G and +3187 G/G genotypes, although significance was not reached. Indeed, the UTR-5 (InsTCCTG***A***C) and UTR-7 (InsTCATG***A***C) haplotypes, which were associated with low levels of sHLA-G do present the +3187A variation site. The only haplotype presenting +3187G is UTR-1. In fact, by summing the possible effect of each of the known variation sites that may influence HLA-G production, UTR-1 is theoretically the most suitable to produce high HLA-G amounts because it is the only UTR that harbors the +3187 G allele, providing greater mRNA stability by modifying the AU-rich motif [15]. In fact, in the present study, UTR-1 was the only haplotype undoubtedly associated with higher levels of sHLA-G.

Since the 14-bp Ins/Del, +3142 C/G and +3187 A/G polymorphic sites have been reported to be associated with the expression levels of the *HLA-G* gene, and since these polymorphic sites are in linkage disequilibrium, the influence of these variation sites might be reciprocal. To test this hypothesis, we compared the levels of sHLA-G according to the simultaneous presence of variation sites associated with high production (14 bp Del/+3142C/+3187G variation sites) or with low production (14 bp Ins/ +3142G/ +3187A). Indeed, 3’UTR haplotypes associated with lower production of sHLA-G as UTR-5 and UTR-7 contained the 14 bp Ins, +3142G and +3187A variation sites. In addition, UTR-8 also contains these polymorphic sites; however, the frequency of this haplotype was very low in both the Brazilian and French populations, impairing further analysis. In contrast, other 3’UTRs exhibiting the 14 bp Del/+3142 C/+3187 G variation sites, like UTR-1, UTR-3, UTR-4, UTR-6, were associated with high or intermediate soluble levels of HLA-G.

 Interestingly, in patients with systemic lupus erythematosus, the UTR-1 haplotype that contains all variation sites reported to be associated with high production of HLA-G has also been associated with protection against disease development [31]. Considering that HLA-G expression in autoimmune disorders may decrease the hyperactivity of the immune system, high expression alleles would be beneficial, whereas low expression ones would be detrimental. 

Besides the 14-bp Ins/Del, +3142 C/G and +3187 A/G, in this study we observed that other polymorphic sites located at the *HLA-G* 3’ UTR were also associated with the levels of sHLA-G, including +3010 C/G, +3027 A/C and +3035 C/T genotypes. The mechanisms by which these variation sites are related to the magnitude of expression of HLA-G have not been studied. Individuals exhibiting the +3010 C/C, +3027 C/C, +3035C/C and C/T genotypes showed significantly higher levels of soluble HLA-G compared to the respective counterpart genotypes. The presence of these variation sites at 3’ UTR haplotypes associated with low [UTR-5 (InsTCCTGAC) and UTR-7 (InsTCATGAC)] or high/intermediate levels of soluble HLA-G [UTR-1 (DelTGCCCGC), UTR-3 (DelTCCCGAC), UTR-4 (DelCGCCCAC), UTR-6 (DelTGCCCAC) and UTR-2 (InsTCCCGAG)] is highly variable. Whether these variation sites exert their influence *per se* or are influenced by other variation sites at the promoter region in linkage disequilibrium with 3’UTR is a question that needs to be clarified and, certainly, will be a matter for further studies. There are no studies in the literature about the association of these sites with diseases; however, our group reported an increased frequency of the +3010 C allele in patients with systemic lupus erythematosus [31].

Overall, UTR-1, which is the most frequent 3’UTR haplotype, was associated with increased HLA-G levels, whereas UTR-5 and UTR-7, which are less frequent ones, were associated with decreased production of sHLA-G. This finding may also have an evolutionary connotation, since UTR-5 and UTR-7 have been quoted as the precursors of HLA-G 3’UTR, and the other UTRs as the modern counterparts [11,53]. Considering that the low frequent UTR-5 and UTR-7 are associated with low levels of sHLA-G and, supposedly, associated with higher rates of miscarriages, the emergence of mutated 3’UTRs associated with higher HLA-G production may have been maintained along human evolution as an advantageous region to keep a more propitious placental environment for normal gestations.

The Janus face HLA-G molecule may exert beneficial or harmful effects depending on the underlying condition. In physiological situations, the constitutive or neoexpression of HLA-G shall protect tissues against damage by immune system cells, including placental, pancreas, thymus, and digestive tract expression. In pathological conditions, in which a vigorous immune response is not desirable, such as in allotransplantation and in autoimmune disorders, the neoexpression of HLA-G is beneficial. In tumor cells or virus-infected cells, the neoexpression of HLA-G shall be highly undesirable due to the inhibition of the immune response. In all of these situations, the identification of individuals genetically prone to differentially express HLA-G may be of help for the definition of novel strategies to control the immune response against the underlying disorder as well as to adapt current therapies regarding the genetic predisposition of patients to produce sHLA-G. 
